# Beyond similarities: overall survival and prognostic insights from [¹⁷⁷Lu]Lu-DOTATOC therapy in neuroendocrine tumors

**DOI:** 10.1007/s00259-025-07221-2

**Published:** 2025-03-28

**Authors:** Tristan Ruhwedel, Julian Rogasch, Imke Schatka, Markus Galler, Peter Steinhagen, Christoph Wetz, Holger Amthauer

**Affiliations:** 1 Department of Nuclear Medicine, Charité– Universitätsmedizin Berlin, Corporate Member of Freie Universität Berlin and Humboldt-Universität zu Berlin, Augustenburger Platz 1, 13353 Berlin, Germany; 2 Department of Gastroenterology, Charité– Universitätsmedizin Berlin, Corporate Member of Freie Universität Berlin and Humboldt-Universität zu Berlin, Augustenburger Platz 1, 13353 Berlin, Germany

**Keywords:** Neuroendocrine tumor, Peptide receptor radionuclide therapy, Overall survival, [^177^Lu]Lu-DOTATOC, RECIST 1.1, Prognostic factor

## Abstract

**Purpose:**

Therapy with [^177^Lu]Lu-DOTATATE is well established for neuroendocrine tumors (NET), but its production generates [^177m^Lu], raising concerns about waste disposal due to its longer half-life. In contrast, [^177m^Lu] is not formed during [^177^Lu]Lu-DOTATOC production. However, data on overall survival (OS) and prognostic factors for [^177^Lu]Lu-DOTATOC remain limited, and its efficacy compared to [^177^Lu]Lu-DOTATATE is uncertain. This study aimed to analyze OS and radiological response in NET patients treated with [^177^Lu]Lu-DOTATOC.

**Methods:**

Monocentric, retrospective analysis of 141 patients with NET (grading: 21% G1, 71% G2, 4% G3, 4% grading unknown; primary: 48% small intestine (SI-NET); 27% pancreas (P-NET); 9% colon/rectum; 1% stomach, 7% lung; 9% CUP-NET) receiving PRRT with [^177^Lu]Lu-DOTATOC. Cox and logistic regression were used to identify prognostic factors for OS or risk of primary progression.

**Results:**

Death from any cause was observed in 85 of 141 patients (60.3%). Median OS was 55.2 months (SI NET G1-G2: 62.7 months; P-NET G1-G2: 41.2 months; NET G3: 26.3 months). Multivariable Cox regression identified baseline De Ritis Ratio (*p* < 0.001), ALP (*p* < 0.001), CgA (*p* < 0.001) and prior therapy with mTOR-inhibitors (*p* = 0.005) as significant prognostic factors of OS. Overall response rate was 12% and disease control rate was 72%. In multivariable logistic regression, primary tumor location (*p* = 0.04) and CgA (*p* = 0.01) were significant prognostic factors for higher risk of primary progression.

**Conclusion:**

The analysis of OS from routine clinical practice shows that PRRT with [^177^Lu]Lu-DOTATOC is an effective treatment option for NET patients, while generating minimal [^177m^Lu]. The evaluated prognostic factors could help to identify patients who particularly benefit from shorter follow-up intervals.

## Introduction

The incidence of neuroendocrine tumors (NET) has significantly increased over recent decades [1]. In the U.S., the rates rose from 4.90 new cases per 100,000 people in 2000 to 8.19 by 2018 [1]. This marked rise underscores the growing need for effective treatments for this rare type of tumor.

According to the current guidelines of the European Society of Medical Oncology (ESMO), peptide receptor radionuclide therapy (PRRT) with either [^177^Lu]Lu-labelled DOTA^0^-Tyr^3^-octreotide (DOTATOC) and DOTA^0^-Tyr^3^-octreotate (DOTATATE) has emerged as a second- to third-line therapy in patients with gastoenteropancreatic NET G_1 − 3_ [2]. The NETTER-1 study, the first randomized controlled phase III study comparing [^177^Lu]Lu-DOTATATE with long-acting octreotide, reported a median overall survival (OS) of 48 months (95% CI: 37.4–55.2 months) in the cohort receiving [^177^Lu]Lu-DOTATATE [3].

Preliminary results from the NETTER-2 study suggest that PRRT is effective for advanced G_2 − 3_ GEP-NET and are suggested to increase the demand for the therapy [4, 5]. Although DOTA-conjugates DOTATATE and DOTATOC are comparable in many ways, with similar somatostatin receptor (SSR) affinity, it is necessary to evaluate OS data to ensure therapeutic effectiveness. However, to date, results of a randomized prospective study testing the therapeutic efficacy of [^177^Lu]Lu-DOTATOC are still lacking. Therefore, data on outcomes of [^177^Lu]Lu-DOTATOC are highly warranted.

Currently, according to the ENETS consensus guidelines only the Ki-67 proliferation marker and serum levels of Chromogranin A (CgA) are assessed as standard prognostic factors in NET patients undergoing PRRT [6, 7]. Recently, the NETTER-1 sub-analysis revealed the presence of a large tumor lesion as a significant prognostic factor of progression-free survival (PFS) in patients receiving PRRT [8]. However, the hepatic tumor burden as well as the baseline alkaline phosphatase showed no significant prognostic value [8]. While these factors provide valuable insights, there remains a significant gap in prognostic markers for OS and radiological outcomes based on RECIST 1.1 criteria. Such markers would be critically important and beneficial for clinicians, particularly given the multiple therapeutic options available in first-line treatment scenarios. The primary objective of this retrospective analysis was to evaluate the OS and radiological outcome according to RECIST 1.1 criteria during PRRT with [^177^Lu]Lu-DOTATOC at our center [9]. Additionally, we evaluated the elaborate prognostic values from recent studies, applying them to our patient cohort to assess their relevance to OS and RECIST 1.1 outcomes.

## Materials and methods

### Study design

This is a monocentric, retrospective analysis of 141 patients with histologically proven NET who received PRRT between September 2007 and August 2021. The following inclusion criteria were defined: (1) histologically proven, metastasized NET, (2) detection of progressive disease (PD) prior to PRRT (3) SSR expression above liver background (e.g. Krenning-Score ≥ 3) in functional imaging (positron emission tomography/computed tomography (PET/CT) or SSR scintigraphy) [10], (4) PRRT performed with [^177^Lu]Lu-DOTATOC (5) follow-up after application of the first cycle PRRT ≥ 12 months.

This analysis was conducted in accordance with the guidelines of the Declaration of Helsinki and approved by the Institutional Ethics Committee. Informed consent was obtained from all subjects involved in the study.

### [^177^Lu]Lu-DOTATOC-PRRT and treatment response evaluation

PRRT was performed with a median of 3 cycles (range: 1–6 cycles) and a scheduled dose of 200 mCi (7.40 GBq) [^177^Lu]Lu-DOTATOC per cycle (radiochemical purity > 95%; pH value 5.0–8.0; endotoxin content < 20 IU/m). PRRT at our center was performed as previously described [5, 11, 12]. Briefly, after two cycles of PRRT, all patients at our center received interim staging to assess treatment response. In the event of clinical progressive disease, interim staging was performed anytime earlier to objectify disease progression. An interdisciplinary tumor board generally confirmed the presence of PD. PRRT was discontinued in the event of PD. Post-therapeutic follow-up imaging was performed every 3 to 6 months. Contrast-enhanced (CE)-CT or alternatively CE-magnetic resonance imaging (MRI) were used for morphological evaluation.

### Endpoints and outcome measures

Blood biomarkers were determined within 4 weeks prior to the first cycle of PRRT. De Ritis ratio (AST/ALT) was defined as previously published [11, 13]. OS was defined as the time from the first cycle of PRRT until death from any cause. Hepatic tumor burden and presence of a large lesion in baseline imaging were assessed as previously performed by the NETTER-1 sub-analysis [8]. Response evaluation was based on the staging performed within 6 months after the last cycle PRRT. The overall response rate (ORR) was calculated by summing partial response (PR) and complete response (CR) according to RECIST 1.1., whereas the disease control rate (DCR) was calculated by summarizing PR, CR and stable disease (SD) [9]. Primary progression was defined as the presence of progressive disease (PD) according to RECIST 1.1 in the staging performed within 6 months after the last cycle PRRT [9].

### Statistical analysis

For statistical analysis, SPSS version 26 (IBM, Chicago, IL, USA) was used. Significance was assumed at ɑ = 0.05. Descriptive values were expressed as median, interquartile range (IQR), and range. Univariable Cox proportional hazards regression for OS was performed including clinical parameters, primary tumor location, pretherapeutic laboratory values, localization of metastases and treatment history before PRRT. Univariable logistic regression was used to identify prognostic factors associated with a higher risk of primary progression, utilizing the same parameters as in the univariable Cox regression analysis. Due to the wide range of CgA values in our cohort, CgA was binarized before inclusion in the analysis. The chosen cut-off of > 204 µg/L reflects twice the upper limit of the normal range for the assay employed and thus defines ‘elevated’ CgA [11, 14, 15]. Additionally, the ALP was binarized according to the cut-off (> ULN) used in the recent NETTER-1 sub-analysis [8]. The hazard ratio (HR) and the 95% confidence interval of the HR were determined for each parameter. All variables with *p* ≤ 0.1 in univariable Cox regression / univariable logistic regression were also candidates for stepwise inclusion into multivariable Cox regression / multivariable logistic regression (criterion: likelihood ratio). The proportional hazard assumption was tested using the goodness-of-fit test and fulfilled by each variable. The Kaplan–Meier method was used to estimate survival rates and median OS.

## Results

### Overall survival

**Fig. 1:**
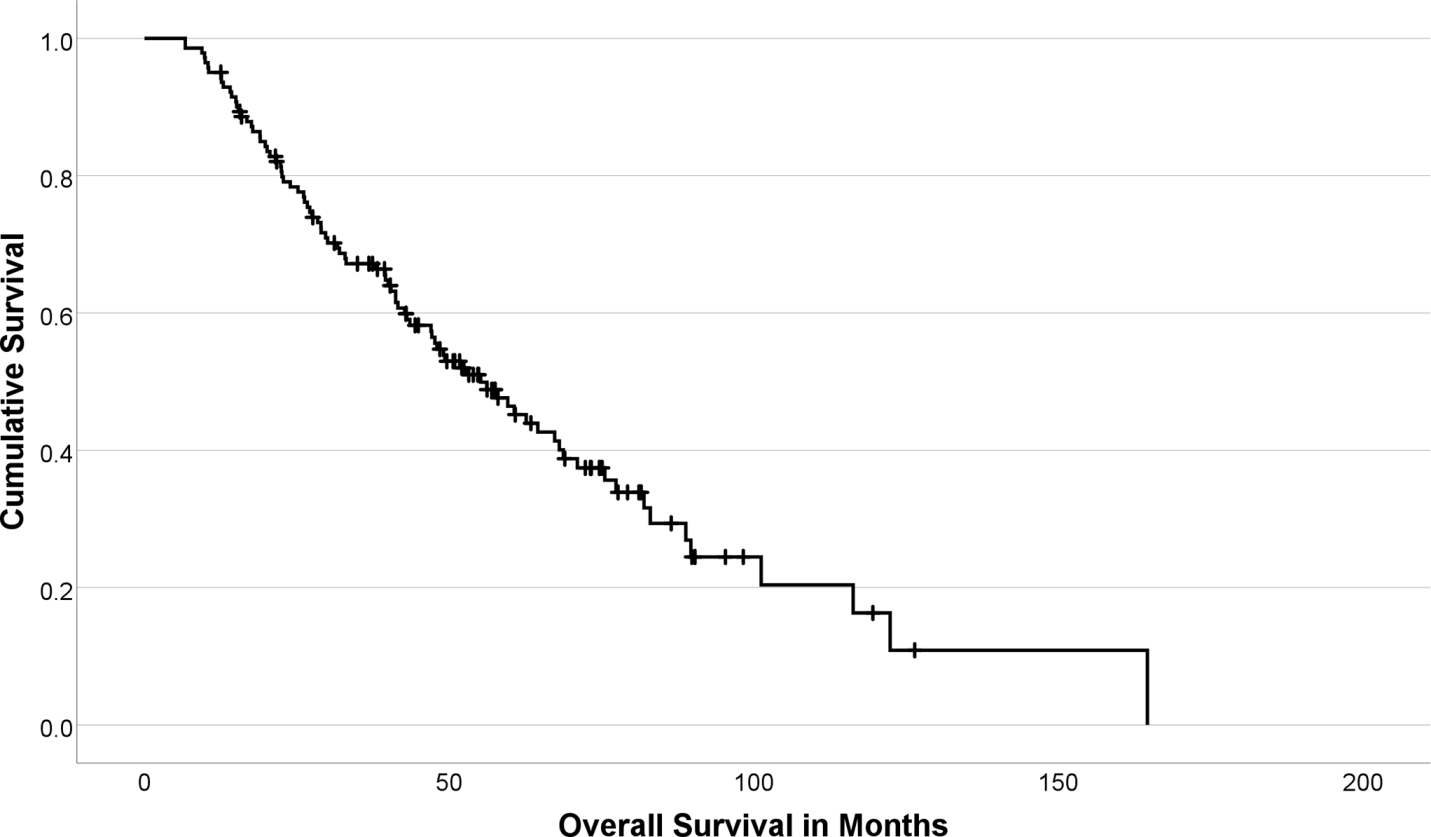
Kaplan-Meier curve of overall survival

Death from any cause was observed in 85/141 patients during follow-up (60.3%). The median OS was 55.2 months for the total cohort (IQR: 27.1–89.7 months; Fig. 1). In patients without observed death, the median follow-up duration was 54.3 months (IQR: 41.0–74.3 months). In GI-NET G_1 − 2_ median overall survival was 62.7 months (IQR: 40.5–101.2 months), while it was 41.2 months (IQR: 17.6–75.6 months) in P-NET G_1 − 2_ and 26.3 months (IQR: 20.6 months– not reached) in NET G_3_. No nephrotoxicity grade ≥ 3, hematologic toxicity grade ≥ 3, tumor lysis syndrome, or dose-limiting liver damage were observed. Patient characteristics are shown in the Appendix (Table A1).

In univariable Cox regression, patients with P-NET (*p* = 0.01) and Lung-NET (*p* = 0.02) had a significantly impaired OS compared to patients with GEP-NET. Regarding pretherapeutic laboratory parameters, a higher De Ritis ratio (*p* < 0.001), a CgA > 204 µg/L (*p* < 0.001) as well as ALP > ULN (*p* < 0.001) were significant prognostic factors for OS. Furthermore, patients with bone metastases (*p* = 0.04), higher liver tumor burden (*p* < 0.001) as well as those with a large lesion in baseline imaging (*p* = 0.01) and those who received a mTOR-inhibitor before PRRT (*p* = 0.03) had a significantly shorter OS (Table 1).

**Table 1:** Univariable Cox regression

Variable	Hazard Ratio	95% Confidence Interval	*p*-Value
**Primary Tumor Location**			0.03
Gastrointestinal (GI-NET)	-	*reference*	-
Pancreatic (P-NET)	1.87	1.13–3.09	**0.01**
Pulmonary (Lung-NET)	2.37	1.14–4.90	**0.02**
CUP	1.64	0.82–3.30	0.16
Grading	0.82	0.51–1.33	0.42
Functionality	0.89	0.56–1.40	0.60
Hedinger Syndrome	1.91	0.69–5.25	0.21
**Baseline Laboratory Values**			
De Ritis Ratio	2.70	1.68–4.36	**< 0.001**
Chromogranin A (in µg/L)	1.00	1.00–1.00	0.84
Chromogranin A (> 204 µg/L)	4.15	2.37–7.25	**< 0.001**
Alkaline Phosphatase (> ULN)	3.06	1.82–5.15	**< 0.001**
**Metastatic Spread**			
Liver	0.57	0.21–1.58	0.28
Lymph Node	1.39	0.76–2.57	0.29
Bone	1.57	1.02–2.42	**0.04**
Lungs	0.45	0.14–1.45	0.18
Peritoneum	1.01	0.57–1.80	0.97
Liver Tumor Burden	1.85	1.34–2.56	**< 0.001**
Presence of a Large Lesion	2.01	1.16–3.49	**0.01**
**Previous Therapy**			
Somatostatin Analogues	0.78	0.48–1.27	0.32
mTOR-Inhibitor	1.91	1.06–3.47	**0.03**
Tyrosine Kinase Inhibitor	0.88	0.21–3.79	0.87
Chemotherapy	1.37	0.86–2.17	0.19
Local Ablative Therapy	1.48	0.71–3.11	0.30
Radiation Therapy	1.09	0.34–3.48	0.88
Transcatheter Arterial Chemoembolization	0.81	0.26–2.57	0.72

Significant values are highlighted in bold

In multivariable Cox regression, higher pretherapeutic levels of De Ritis Ratio (HR: 2.98; *p* < 0.001), ALP > ULN (HR: 2.72; *p* < 0.001) as well as pretherapeutic CgA > 204 µg/L (HR: 4.96; *p* < 0.001) and therapy with mTOR-inhibitors before PRRT (HR: 2.04; *p* = 0.026) were independent prognostic factors of shorter OS (Table 2).

**Table 2:** Multivariable Cox regression

Variable	Hazard Ratio	95% Confidence Interval	*p*-Value
**Primary Tumor Location**			0.11
Gastrointestinal (GI-NET)	-	*Reference*	-
Pancreatic (P-NET)			**0.04**
Pulmonary (Lung-NET)			0.57
CUP			0.43
**Pretherapeutic Laboratory Values**			
De Ritis Ratio	2.98	1.85–4.79	**< 0.001**
Chromogranin A (> 204 µg/L)	4.96	2.72–9.06	**< 0.001**
Alkaline Phosphatase (> ULN)	2.72	1.57–4.69	**< 0.001**
**Metastatic Spread**			
Bone			0.18
Liver Tumor Burden			0.18
Presence of a Large Lesion			0.39
**Previous Therapy**			
mTOR-Inhibitor	2.04	1.09–3.81	**0.026**

Significant values are highlighted in bold

**Fig. 2:**
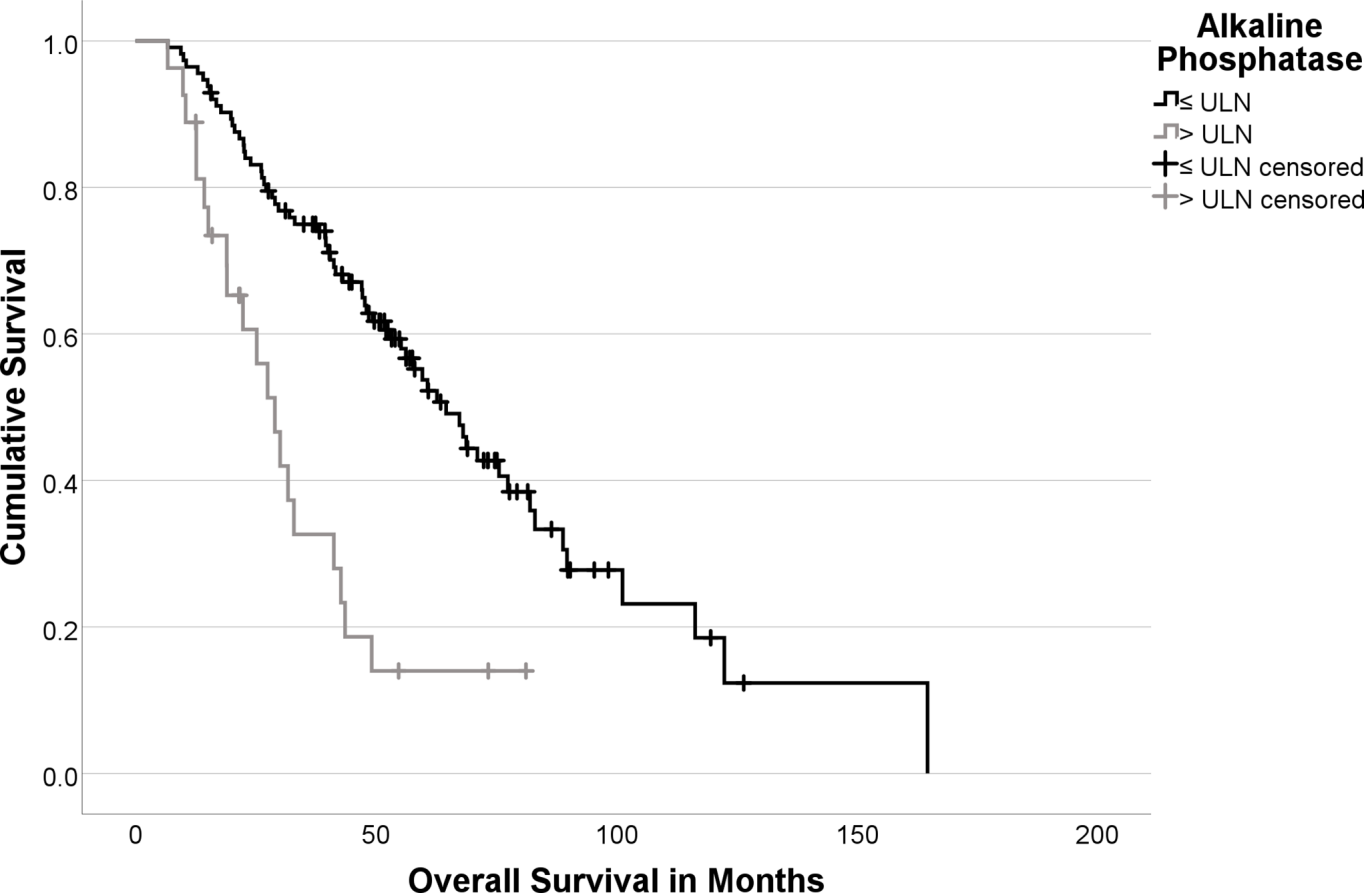
Kaplan-Meier Curves Stratified by ALP > ULN (Logrank: *p* < 0.001)

Figure 2 displays the Kaplan-Meier curves stratified by baseline ALP levels.

**Fig. 3:**
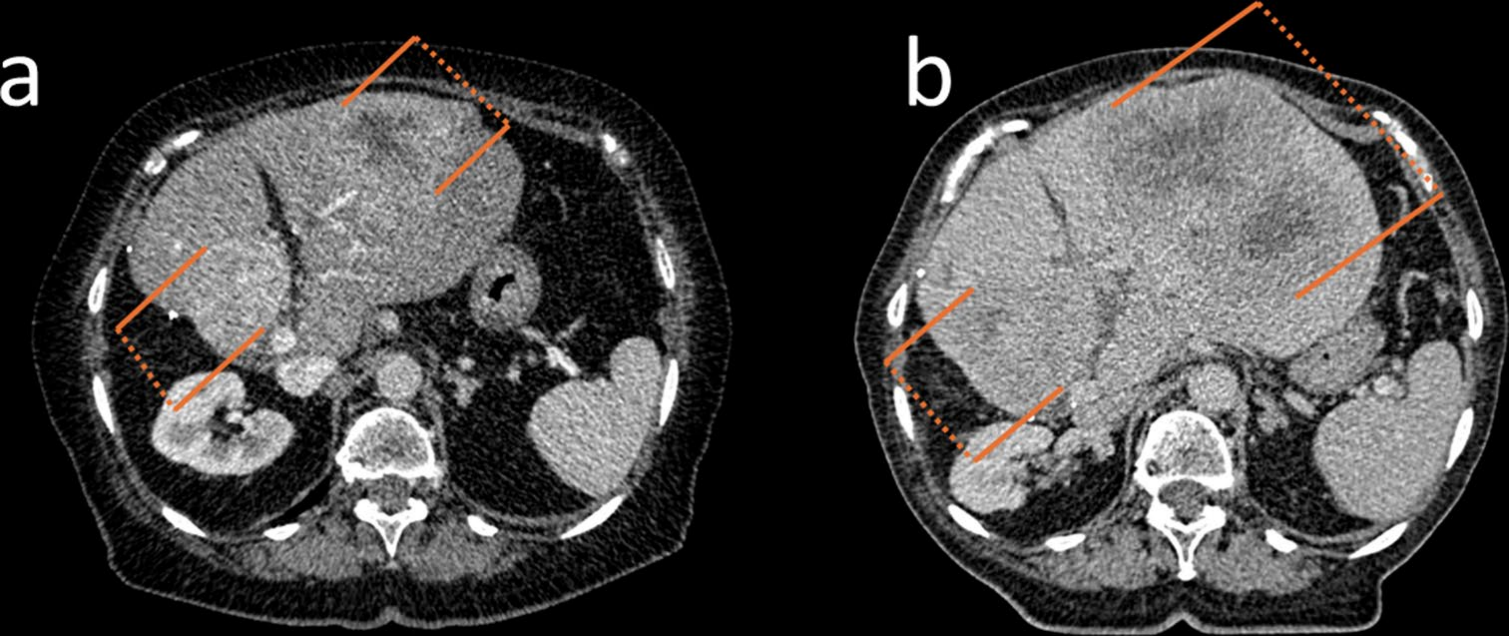
CT-images at baseline (**a**) and after 4 cycles of PRRT (**b**) of a representative patient with elevated risk factors at baseline (De Ritis ratio: 1.43, CgA: 12.350 µg/L, and ALP: 120 U/L), who suffered from primary progression and showed an OS of 19 months

**Fig. 4:**
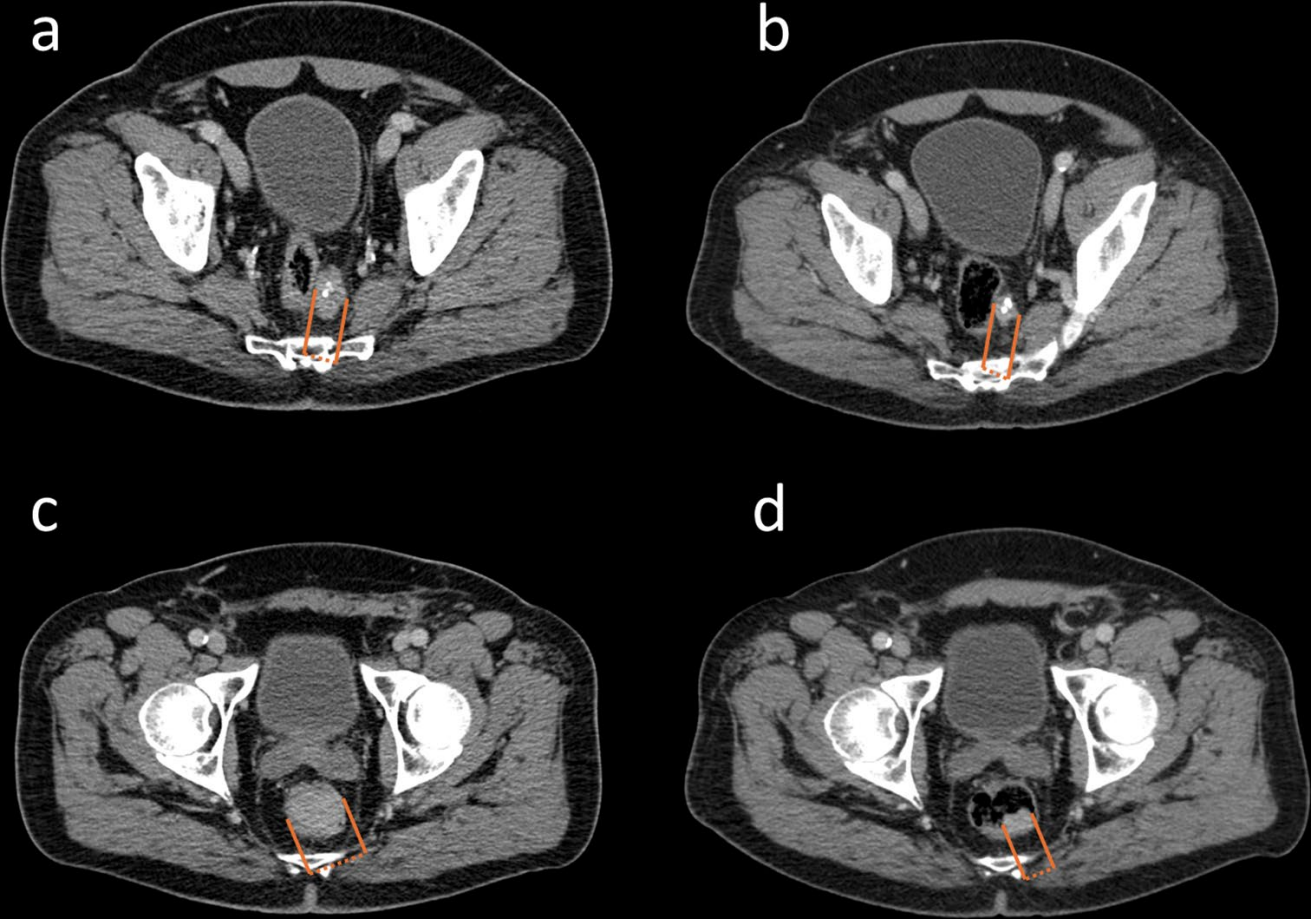
CT-images at baseline (**a**/**c**) and after 4 cycles of PRRT (**b**/**d**) of a patient with favorable risk factors at baseline (De Ritis ratio: 0.63, CgA: 14 µg/L, and ALP: 63 U/L), who had a partial response to PRRT and showed an OS of 64 months

Fig. 3 and 4 illustrate patients with favorable as well as elevated baseline parameters showing differences in OS and response to therapy

### RECIST 1.1

After PRRT, RECIST 1.1 outcomes were available in 123 patients. We observed PD in 35 patients (29%), SD in 73 (60%), PR in 14 (11%), and CR in only 1 (1%). ORR was 12% and DCR was 72%.

When performing univariable logistic regression, Lung-NET in comparison to GEP-NET (*p* = 0.02) and a pretherapeutic CgA > 204 µg/L (*p* = 0.007) were significant prognostic factors for a higher risk for primary progression. Primary tumor location (*p* = 0.06) and the presence of bone metastases (*p* = 0.053) showed a trend towards higher risk for primary progression (Table 3).

**Table 3:** Univariable logistic regression

Variable	Odds Ratio	95% Confidence Interval	*p*-Value
**Primary Tumor Location**			0.06
Gastrointestinal (GI-NET)	-	*reference*	-
Pancreatic (P-NET)	1.11	0.45–2.73	0.81
Pulmonary (Lung-NET)	5.79	1.32–25.45	**0.02**
CUP	0.26	0.03–2.18	0.22
Grading	0.95	0.42–2.11	0.89
Functionality	0.87	0.38–1.98	0.73
Hedinger Syndrome	0.51	0.06–4.56	0.51
**Pretherapeutic Laboratory Values**			
De Ritis Ratio	1.15	0.47–2.81	0.76
Chromogranin A (in µg/L)	1.00	1.00–1.00	0.92
Chromogranin A (> 204 µg/L)	4.13	1.48–11.58	**0.007**
Alkaline Phosphatase > ULN	2.13	0.85–5.34	0.11
**Metastatic Spread**			
Liver	1.94	0.22–17.24	0.55
Lymph Node	0.86	0.30–2.46	0.78
Bone	2.17	0.99–4.74	0.053
Lungs*	n/a	n/a	n/a
Peritoneum	1.07	0.38–3.00	0.90
Liver Tumor Burden	1.18	0.67–2.09	0.57
Presence of a Large Lesion	0.96	0.39–2.33	0.93
**Previous Therapy**			
Somatostatin Analogues	0.61	0.26–1.44	0.26
mTOR-Inhibitor	1.70	0.57–5.08	0.34
Tyrosine Kinase Inhibitor*	n/a	n/a	n/a
Chemotherapy	1.50	0.66–3.39	0.33
Local Ablative Therapy	2.90	0.79–10.71	0.11
Radiation Therapy	1.80	0.29–11.27	0.53
Transcatheter Arterial Chemoembolization	1.06	0.20–5.72	0.95

Significant values are highlighted in bold. *For this variable no values could be calculated, due to complete separation in this cohort

In multivariable logistic regression, Lung-NET compared to GI-NET (*p* = 0.04), as well as a pretherapeutic CgA > 204 µg/L (*p* = 0.01) remained significant prognostic factors for a higher risk of primary progression (Table 4).

**Table 4:** Multivariable logistic regression

Variable	Odds Ratio	95% Confidence Interval	*p*-Value
**Primary Tumor Location**			0.09
Gastrointestinal (GI-NET)	-	*reference*	-
Pancreatic (P-NET)	1.26	0.50–3.22	0.62
Pulmonary (Lung-NET)	4.86	1.06–22.24	**0.04**
CUP	0.28	0.02–1.96	0.18
**Pretherapeutic Laboratory Values**			
Chromogranin A (> 204 µg/L)	4.07	1.42–11.67	**0.01**
**Metastatic Spread**			
Bone			0.23

Significant values are highlighted in bold

## Discussion

We described prognostic factors for OS in a cohort of 141 patients with NET G_1 − 3_ treated with [^177^Lu]Lu-DOTATOC. Additionally, RECIST 1.1 outcomes were reported. Patients had a median OS of 55.2 months from the start of PRRT, while DCR and ORR were 72% and 12%, respectively. Furthermore, we investigated prognostic factors for both OS and primary progression and found a significant and independent prognostic value for OS of the De Ritis ratio, CgA and ALP before PRRT as well as previous treatment with mTOR-inhibitors. In contrast, elevated pretherapeutic levels of CgA and the presence of a pulmonary primary had an independent, significant prognostic value for the risk of primary progression.

Interestingly, our cohort showed a higher median OS in SI-NET G_1–2_ compared to the NETTER-1 study (62.7 vs. 48 months), despite including fewer G_1_ tumors (23% vs. 66%) and more G_2_ tumors (77% vs. 34%) [3, 16]. Differences may arise because our study covered a broader time period, during which both diagnostic and treatment of NETs may have evolved. For example, while the NETTER-1 study evaluated SSR expression using SSR-scintigraphy with OctreoScan, our cohort gradually transitioned from SSR scintigraphy to the more sensitive SSR-PET/CT, likely improving patient selection for PRRT [17]. Additionally, advancements in therapies might have contributed to the higher OS observed in our study. However, other retrospective studies found also higher median OS values of 61 and 60 months, in patients also receiving [^177^Lu]Lu-DOTATATE [18–20], while an analysis of patients receiving [^177^Lu]DOTATOC published in 2015 found a median OS of 45.5 months [21]. In a recently published study by Luna-Gutiérrez et al. on 53 patients with GEP-NET treated with [^177^Lu]DOTATOC, the authors reported a median overall survival (OS) of 44.2 months [20]. However, the 95% confidence interval is not provided and due to the small study population differences to our result may have arisen by chance. Therefore, data on median OS in patients receiving [^177^Lu]Lu-DOTATOC is urgently needed.

Regarding DCR and ORR according to RECIST 1.1, a recent meta-analysis performed by Wang et al., which included 1,127 patients with NET receiving PRRT, reported a ORR of 10– 69% and a DCR of 68– 94% [22]. The wide range of ORR results was explained by significant heterogeneity between the studies [22]. Additionally, comparison to our analysis is only possible to a limited extend as Wang et al. included both PRRT with [^177^Lu]Lu-DOTATOC and [^177^Lu]Lu-DOTATATE and did not separate the results [22]. Furthermore Baum et al. reported of a cohort of 65 patients with NET treated with [^177^Lu]Lu-DOTATOC with an ORR of 33.9% and a DCR of 66.1% [23]. However, detailed information on when the outcomes were measured was not provided, as patients underwent imaging before each PRRT cycle and during restaging every 7 months after the last cycle. Additionally, Luna-Gutiérrez found an ORR of 52% and a DCR of 97% in 38 patients with GEP-NET treated with [^177^Lu]Lu-DOTATOC, but a much lower ORR and DCR of 0% and 71%, respectively, in additional 19 patients with Non-GEP-NET [20]. Considering the limited data on RECIST 1.1 outcomes in patients receiving [^177^Lu]Lu-DOTATOC and the variability in study designs, our study provides valuable insights into the efficacy of therapy with [^177^Lu]Lu-DOTATOC.

Overall, these results suggest that [^177^Lu]Lu-DOTATOC is an effective alternative to [^177^Lu]Lu-DOTATATE. The ongoing COMPETE (NCT03049189) and COMPOSE (NCT04919226) trials are expected to provide much-needed information on outcomes with [^177^Lu]Lu-DOTATOC and could redefine the role of PRRT in the treatment sequence for NET. However, [^177^Lu]Lu-DOTATOC and [^177^Lu]Lu-DOTATATE do not have identical receptor affinities, as DOTATATE-based ligands generally present a higher affinity for SSR subtype 2, which is commonly overexpressed in NET [24]. In particular, Esser et al. showed a longer tumor residence time, when [^177^Lu]Lu-DOTATATE was administered [25]. In contrast, Poeppel et al. compared [^68^Ga]Ga-DOTATATE and [^68^Ga]Ga-DOTATOC in patients receiving both PET radiopharmaceuticals and concluded that [^68^Ga]Ga-DOTATOC might have an pharmaceutical advantage, as the SUVmax across all lesions was significantly higher [26]. Meanwhile, Schuchardt et al. did not show a significant difference in tumor absorbed dose between [^177^Lu]Lu-DOTATATE and [^177^Lu]Lu-DOTATOC [27]. However, they noted an intrapatient variability with a lower tumor absorbed dose when [^177^Lu]Lu-DOTATOC was administered after [^177^Lu]Lu-DOTATATE, although the mean interval of 18 months between both cycles complicates a direct comparison, given the possibility of a tumor progression in the interim. Ultimately, a study directly comparing [^177^Lu]Lu-DOTATOC and [^177^Lu]Lu-DOTATATE is needed to determine whether these differences in receptor affinity translate into a measurable clinical impact, especially if [^177^Lu]Lu-DOTATOC receives approval for NET by both FDA and EMA in the future.

Due to these differences of both radiopharmaceuticals, clinically relevant variations in prognostic and predictive factors may theoretically arise. In a subanalysis of the well-known NETTER-1 cohort, the presence of a large tumor lesion was found to be a significant prognostic factor for PFS in patients undergoing PRRT with [^177^Lu]Lu-DOTATATE [8]. In contrast, an altered ALP at baseline was not found to be statistically significant [8]. Unfortunately, since a multivariable analysis was not performed, adjustment for potential confounders was missing. Additionally, patients receiving [^177^Lu]Lu-DOTATATE showed low event rates of 15.5% in the group with ALP ≤ ULN and 19.5% in the group with ALP > ULN. Therefore, median PFS was not reached at the time of analysis, potentially leading to low statistical power. In contrast, baseline ALP showed significant prognostic value for OS in a study of 91 NET patients receiving four cycles of [^177^Lu]Lu-DOTATATE, performed by Akhavanallaf et al. [28], as well as in another retrospective study including patients with P-NETs (*n* = 102) receiving [^177^Lu]Lu-DOTATATE [29]. Recently, the baseline De Ritis ratio and its prognostic significance for PFS in patients with NET undergoing both initial PRRT and salvage PRRT were analyzed [11, 30]. However its prognostic value for PFS and OS in patients receiving [^177^Lu]Lu-DOTATATE has not yet been fully established. Nonetheless, an elevated pretherapeutic CgA level was also a significant prognostic factor for OS in patients receiving [^177^Lu]Lu-DOTATATE [28, 31, 32]. Regarding other significant prognostic factors in our study cohort, pretreatment with mTOR-inhibitors might be an indirect sign of a higher tumor proliferation rate or of a pancreatic primary. According to the current ESMO-guidelines, it is regularly used prior to PRRT in small intestinal NET (SI-NET) G2 as well as P-NET G1-G3, while SI-NET G1 receive PRRT prior to mTOR-inhibitors [2]. However, the grading showed no prognostic value in both univariable Cox regression as well as logistic regression. Conversely, the presence of a higher number of therapy lines before PRRT was also a significant prognostic factor for OS in patients receiving [^177^Lu]Lu-DOTATATE [28]. As indicated, available data comes mainly from retrospective studies with varying study designs, endpoints and patients populations, complicating any direct comparison between [^177^Lu]Lu-DOTATATE and [^177^Lu]Lu-DOTATOC. Therefore, only a limited comparison of prognostic factors is possible due to the absence of prospective, head-to-head trials, which makes it difficult to draw practical conclusions about truly predictive differences between both radiopharmaceuticals.

It is important to recognize that the production of ^177^Lu can result in the formation of metastable ^177m^Lu, a long-lived isotope with a half-life of 161 days. The presence of ^177m^Lu in [^177^Lu]Lu-DOTATATE necessitates more rigorous waste disposal procedures. To address the challenges associated with metastable lutetium and simplify waste management, [^177^Lu]Lu-DOTATOC can be employed as an alternative. Its production method minimizes the generation of long-lived radioactive contaminants, thereby reducing the need for specialized disposal measures [33].

Limitations may arise due to the retrospective setting of our current analysis. The predictive capability of the prognostic factors could not be evaluated due to the absence of a control group of patients receiving different treatment. Consequently, based on our current data, we cannot determine whether patients with impaired prognostic factors would benefit more from other therapies or whether these factors simply reflect a generally worse prognosis. Future investigations should utilize prospective study designs to confirm our preliminary findings and to secure a carefully chosen, homogenous patient cohort. Furthermore, due to the retrospective design baseline and follow-up stagings were conducted using varying imaging modalities– e.g. CE-MRI versus CE-CT, and SSTR-PET versus SSTR scintigraphy, and different timepoints, potentially limiting the comparability of our findings to those of other studies [5]. Our inclusion period (2007–2021) covers a time of evolving diagnostics and therapies for neuroendocrine tumors. In the beginning, SSTR scintigraphy was the clinical standard, detecting predominantly higher SSTR-expressing tumors, whereas later, more sensitive SSTR-PET/CT was used. This development may introduce a selection bias towards higher SSTR-expressing tumors in earlier years. However, we consistently required SSTR expression above liver background (Krenning score ≥ 3) for treatment with PRRT— whether assessed by scintigraphy or PET/CT [10]. Additionally, due to missing documentation, follow-up treatment after the completion of PRRT was consistently not available and therefore generally not assessed, which may have introduced a confounding bias. As we perform standardly an interim staging at our center, patients received varying numbers of therapy cycles as PRRT was discontinued upon detection of PD [5]. This may lead to a limitation when comparing our results with studies that treated patients with a standardized number of treatment cycles.

## Conclusion

This analysis highlights that [^177^Lu]Lu-DOTATOC is an effective treatment option for patients with NET. We identified significant prognostic factors for OS and the risk of primary progression, providing valuable insights for patient care. Furthermore, these results contribute to the limited outcome data on PRRT with [^177^Lu]Lu-DOTATOC. Notably, therapy with [^177^Lu]Lu-DOTATOC offers advantages in specialized waste disposal, further increasing its practicality over [^177^Lu]Lu-DOTATATE.

## Data Availability

The datasets generated during and analysed during the current study are available from the corresponding author on reasonable request.

## Appendix

**Table A1:** Patient characteristics. (adapted from [12])

Variable	*n* (%) or Median (Range)
Age in years	64 (34–87)
**Sex**	
Men	89 (63%)
Women	52 (37%)
**Primary location**	
Gastrointestinal (GI-NET)	67 (48%)
Pancreatic (P-NET)	38 (27%)
Pulmonary (Lung-NET)	12 (9%)
CUP	10 (7%)
Gastrointestinal (GI-NET)	1 (1%)
Pancreatic (P-NET)	13 (9%)
Metastatic disease	138 (98%)
**Metastatic Spread**	
Liver	135 (96%)
Lymph Node	118 (84%)
Bone	54 (38%)
Lungs	6 (4%)
Peritoneum	24 (17%)
Functionality	46 (33%)
Hedinger Syndrome	6 (4%)
**Grading**	
G1	30 (21%)
G2	100 (71%)
G3	5 (4%)
Unknown	6 (4%)
Ki-67 Index	5 (1–40)
Number of PRRT Cycles	3 (2–6)
**Previous treatment**	
Operative Resection	93 (66%)
Somatostatin Analogues	108 (77%)
mTOR-Inhibitor	17 (12%)
Tyrosine Kinase Inhibitor	4 (3%)
Chemotherapy	41 (29%)
Local Ablative Therapy	12 (9%)
Radiation Therapy	5 (4%)
Transcatheter Arterial Chemoembolization	7 (5%)
